# 胸膜恶性黑色素瘤：1例报告及文献复习

**DOI:** 10.3779/j.issn.1009-3419.2020.101.11

**Published:** 2020-05-20

**Authors:** 博闻 李, 健 胡, 栋 常

**Affiliations:** 100050 北京，首都医科大学附属北京友谊医院胸外科 Department of Thoracic Surgery, Beijing Friendship Hospital, Capital Medical University, Beijing 100050, China

**Keywords:** 恶性黑色素瘤, 胸膜, 胸腔镜, Malignant melanoma, Pleura, Thoracoscopy

## Abstract

恶性黑色素瘤是由人体黑色素细胞产生的肿瘤，其恶性程度高，转移发生早，患者死亡率高，多数恶性黑色素瘤是由人体皮肤黑痣恶变产生的，少数也可见于直肠和肛门等消化道。原发于人体胸膜的恶性黑色素瘤十分罕见，偶见于病例个案报道。现报道1例首都医科大学附属北京友谊医院应用胸腔镜联合病理确诊的以咳嗽、咳痰及胸腔积液为首发症状的恶性黑色素瘤患者的临床资料，并结合文献资料进行分析总结。

## Abstract (en)

Malignant melanoma is a kind of tumor produced by human melanocytes. It has a high degree of malignancy, early metastasis and high mortality. Most melanomas are caused by malignant skin nevus and can also be seen in the digestive tract such as rectum and anus. Primary malignant melanoma of pleura is rare, rarely seen in case reports. This paper reports the clinical data of a case of malignant melanoma with cough, expectoration and pleural effusion as the first symptoms diagnosed by thoracoscopy combined with pathology in Beijing Friendship Hospital Affiliated to Capital Medical University, and analyzes and summarizes the literature data.

现报道1例首都医科大学附属北京友谊医院应用胸腔镜联合病理确诊的以咳嗽、咳痰及胸腔积液为首发症状的恶性黑色素瘤患者的临床资料，并结合文献资料进行分析总结。

## 1 一般资料

### 1.1 患者资料

女，49岁，在编职工。因“咳嗽6个月，咳痰3周，胸闷1日”于2018年9月4日入院。

现病史：患者6个月前无明显诱因出现咳嗽，干咳为主，无发热、咳痰等，患者未予治疗，3周前咳嗽症状加重，伴白色粘痰，易咳出，痰量不多，稍有乏力，无明显发热、畏寒、寒战，无喘息、胸痛，无心悸盗汗。患者1 d前上一层楼后觉胸闷，无出汗、心悸、黑曚，于当地医院行胸部计算机断层扫描(computed tomography, CT)检查示左肺多发结节，右侧大量胸腔积液。

既往史：5年前诊断血脂增高，未行治疗。最近1年因腹胀口服中药治疗，排便2次/d-3次/d。8年前因子宫肌瘤大量出血性子宫切除术，曾行右侧乳腺良性结节切除术，具体时间不详，否认其他既往病史，其他系统回顾无特殊。

个人史：出生并久居于本地，否认疫水、疫区接触史，否认其他放射性物质及毒物接触史，否认吸烟史，否认饮酒史。

查体:体温36.1 ℃，脉搏84次/分(规则)，呼吸20次/分(规则)，血压130/79 mmHg。神志清楚，发育正常，营养中等。全身皮肤及粘膜：色泽正常，未见皮疹、色素痣、瘀点、瘀斑。左颌下可及肿大淋巴结，直径约0.5 cm，活动度可，无压痛；右侧腋窝可及肿大淋巴结，直径约1 cm，活动度可，轻压痛。气管居中，胸廓对称无畸形，左肺叩诊清音，右肺叩诊浊音，左侧呼吸音稍粗，右侧未闻及呼吸音，未闻及异常呼吸音及干湿啰音。心界不大，心率84次/分，节律齐，未闻及心音增强或减弱，未闻及额外心音及杂音，未闻及心包摩擦音。腹平，可见手术瘢痕，无静脉曲张，无异常血管，腹软，无压痛，无反跳痛，肝脾未触及，下腹未及异常包块，叩诊鼓音，移动性浊音阴性，肝区、脾区、肾区无叩痛。肠鸣音存在。脊柱四肢无畸形，四肢活动自如，双下肢无水肿。生理反射存在，病理反射未引出。外阴部皮肤及粘膜颜色正常，未见皮疹、色素痣等。

### 1.2 辅助检查

2018-09-10胸部增强CT (图 1)示：胸廓两侧对称，支气管血管束清晰，双肺及双侧叶间裂可见多发大小不等实性结节影，右侧胸膜呈不均匀或结节状增厚，平扫CT值约19 HU，增强扫描CT值约56 HU。右侧胸膜不均匀增厚并强化，考虑恶性改变，间皮瘤?转移瘤?其他?；双肺多发结节灶，考虑转移瘤；纵隔多发肿大淋巴结，考虑淋巴结转移。

**1:**
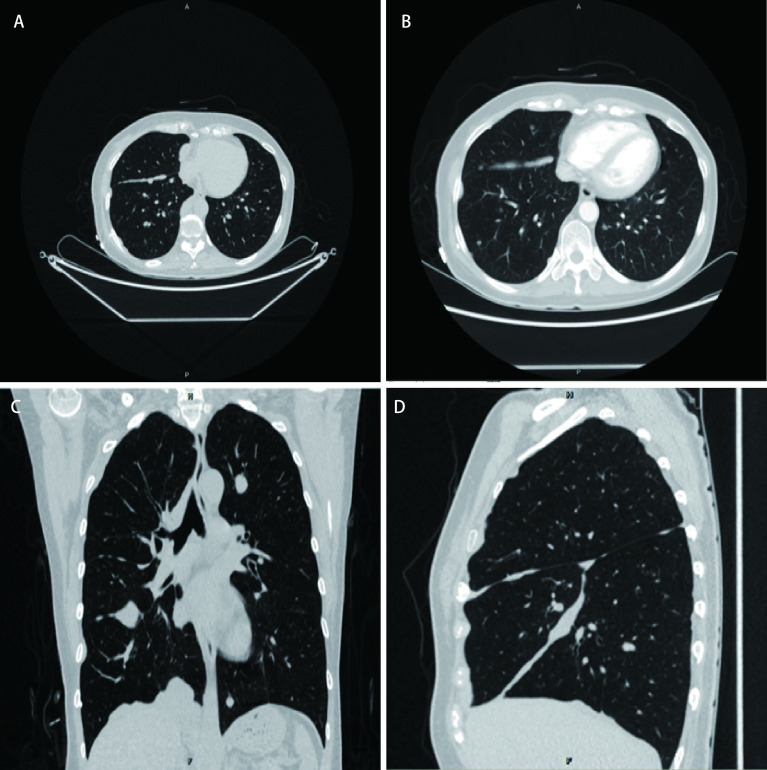
胸部CT平扫+增强示双肺及双侧叶间裂可见多发大小不等实性结节。A：胸部CT平扫右肺叶间裂可见多发大小不等实性结节影，右侧胸膜呈不均匀或结节状增厚；B：与A图同一截面增强后可见叶裂间结节明显强化，右侧胸膜不均匀增厚并强化；C：胸部CT平扫冠状位重建可见双肺多发结节影，纵隔多发肿大淋巴结；D：胸部CT平扫右肺矢状位重建可见右肺水平裂及斜裂间多发大小不等实性结节影，右侧胸膜呈不均匀或结节状增厚 Chest CT plain scan + enhancement shows multiple solid nodules with unequal sizes in bilateral lobes and bilaterally lobed lobes. A: multiple solid nodule shadows with unequal sizes in right lobed lobes and uneven or nodular thickening in right pleura on chest CT plain scan; B: obvious enhancement in the same cross section as A, and uneven thickening and enhancement in right pleura; C: plain scan in chest CT In the coronal reconstruction, multiple nodular shadows of both lungs and swollen lymph nodes of mediastinum can be seen; D: in the sagittal reconstruction of the right lung on plain CT scan of the chest, multiple solid nodular shadows of unequal size can be seen between the horizontal and oblique fissures of the right lung, and the right pleura is uneven or nodular thickening. CT: computed tomography

2018-09-26腹部增强CT (图 2)示：肝脏形态大小如常，轮廓规整，肝实质密度均匀，肝内可见多发大小不等低密度影，大着位于肝S2，截面约2.7 cm × 1.8 cm，CT值约18 HU，部分病灶周围似可见稍高强化，不除外转移瘤可能。动脉期肝实质密度不均匀，平扫及门脉期呈均匀密度。患者腹部CT影像检查结合既往病史，患者最近一年出现反复腹胀及大便次数增多情况，考虑肝内结节为转移病灶。

**2:**
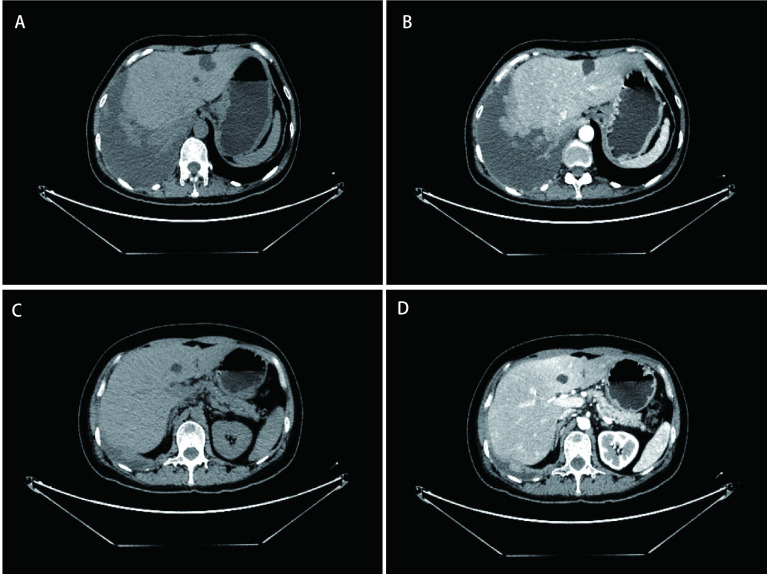
腹部CT平扫+增强示肝内可见多发大小不等低密度影，部分病灶周围似可见稍高强化，不除外转移瘤可能。A、B：腹部CT同一平面平扫与增强对比图像；C、D：腹部CT同一平面平扫与增强对比图像 Plain scan and enhanced scan of abdominal CT showed multiple low-density shadows of different sizes in the liver, and some lesions seemed to have slightly higher enhancement around, excluding the possibility of metastasis. A and B are plain and contrast enhanced images of the same plane of abdominal CT; C and D are plain and contrast enhanced images of the same plane of abdominal CT

入院后完善相关化验检查，2018-09-06血常规、生化检查未见明显异常，D-二聚体1.50 μg/mL较正常值上限偏高，癌抗原125 (cancer antigen 125, CA125) 508.93 U/mL较正常值上限偏高。患者为进一步明确病变性质，于2018-09-05在超声引导下行胸腔穿刺置管术，术后留取胸水标本(图 3)送病理涂片结果示：胸水涂片可见淋巴细胞、增生的间皮细胞及少量非典型的细胞团，未见明确肿瘤细胞。同时每日抽取胸水缓解症状。胸水化验结果回报：淡黄色，微浑，不凝固，李凡它试验阴性，比重 < 1.018，有核细胞计数801×10^6^/L，单个核细胞89%。胸水生化：白蛋白29.7 g/L，总蛋白45.1 g/L，葡萄糖5.92 mmol/L，腺苷脱氨酶(adenosine deaminase, ADA) 6.00 U/L，乳酸脱氢酶(lactate dehydrogenase, LDH) 148 U/L，胸水CA125含量为1, 909.12 U/mL。

**3:**
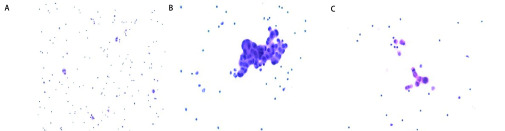
胸水涂片。A、B和C分别为三次胸水涂片病理结果回报，显微镜下放大20倍，均未查见肿瘤细胞 Hydrothorax smear. A, B and C images were the pathological results of three times of pleural effusion smear, respectively. Under the microscope, they were magnified 20 times, and no tumor cells were found

根据患者入院后CT及化验检查，初步诊断考虑肺恶性肿瘤伴胸膜转移，为进一步明确病理学类型并指导治疗，遂转入外科行胸腔镜胸膜活检术。2018-09-13外科胸腔镜(图 4)检查发现：右侧胸腔内大量淡黄色胸水，探查见壁层胸膜及膈肌面大量大小不等的菜花状隆起，白色或呈透明状，部分呈黑色污泥状，触之质脆，易出血，用电刀沿结节基地电凝取壁层胸膜结节送病理检查，术后放胸腔引流管。胸膜结节术后病理检查(图 5)提示恶性黑色素瘤。免疫组化：S-100+；MelaA+；HMB45+；Vimentin+；TTF-1、NapsinA-；Calrentinin-；WT1-；CK-；CK5/6 (2次) -；PR-；GCDFP15-；D2-40-。

**4:**
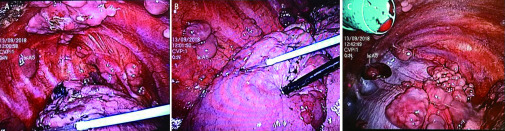
胸腔镜下可见肺表面及胸膜大量大小不等的菜花状隆起。A和B：右侧胸腔胸膜及肺表面大量大小不等的菜花状隆起；C：右侧胸腔膈肌上大量大小不等的菜花状隆起) Under thoracoscopy, a large number of cauliflower like protuberances with different sizes can be seen on the lung surface and pleura. A and B: a large number of cauliflower like protuberances of different sizes on the right pleura and lung surface; C: a large number of cauliflower like protuberances of different sizes on the right thoracic diaphragm

**5:**
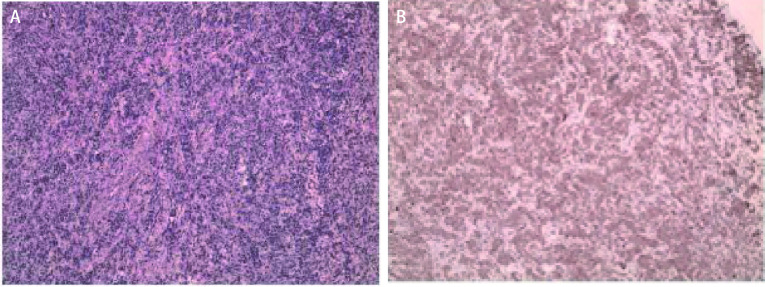
胸膜结节术后病理诊断。A：胸膜结节1枚，显微镜下放大10倍可见单一异型细胞，结合免疫组化，诊断为黑色素瘤；B：胸膜结节1枚，显微镜下放大10倍可见异型细胞，结合免疫组化，诊断为黑色素瘤 Pathological diagnosis of pleural nodule after operation. A: a pleural nodule, with a single heterotypic cell seen at a magnification of 10 times under the microscope. Combined with immunohistochemistry, it was diagnosed as melanoma; B: a pleura nodule with 10 times magnification under the microscope. Combined with immunohistochemistry, it was diagnosed as melanoma

### 1.3 诊断及治疗

结合患者胸腔镜探查、病理检查结果及全身查体结果，确诊患者为原发性胸膜恶性黑色素瘤，予以患者“达卡巴嗪+重组人血管内皮抑制素”化疗联合抗血管生成药物治疗。目前患者生存，间断发热，咳嗽、咳痰。2018年12月复查胸部CT提示：右侧胸膜不均匀增厚，右侧胸膜弥漫性肿瘤，纵隔多发肿大淋巴结，右肺多发结节病变，右侧少量胸腔积液。持续随访中。

## 2 讨论

### 2.1 胸膜恶性黑色素瘤的病因和分类

恶性黑色素瘤是由人体黑色素细胞产生的肿瘤，其恶性程度高，远处转移发生较早，患者的死亡率较高，多数恶性黑色素瘤是由人体皮肤黑痣恶变产生的，少数也可见于直肠和肛门等消化道^[1]^。原发于人体胸膜的恶性黑色素瘤十分罕见，偶见于病例个案报道^[2]^。黑色素瘤主要包括两种亚型，分别起源于黑色素母细胞和黑素细胞，两种亚型都可具有良性或恶性临床病程^[3]^。恶性黑色素瘤是起源于皮肤、黏膜、眼和中枢神经系统色素沉着区域的黑色素细胞的恶性肿瘤，其发病率占全部恶性肿瘤发病率的1%-3%，多见于皮肤，原发于肺部者十分罕见，仅占全部肺部肿瘤的0.01%。目前，针对胸膜恶性黑色素瘤的发病机制研究尚不十分明确。目前主要有以下3种假说：①机体内存在的黑色素细胞在胚胎形成期向表皮或真皮层迁移的同时也可向机体内脏器官迁移，导致食管、咽喉、脑、肺等处同样存在黑色素细胞，位于气管、支气管、咽喉和食管残存的原始成黑色素细胞则均可以进一步分化为原发性肺恶性黑色素瘤；②黏膜下支气管腺的黑色素细胞化生；③起源于下呼吸道中的多潜能干细胞向黑色素细胞分化^[4]^。

### 2.2 临床表现和诊断

胸膜恶性黑色素瘤不论是原发灶还是转移灶都十分罕见，目前国内有文献报道的胸膜恶性黑色素瘤病例约10例。皮肤原发胸膜转移的恶性黑色素瘤患者，通过有针对性的病史采集以及体格检查可能对临床诊断提供帮助。原发于胸膜的恶性黑色素瘤患者由于临床症状和体征均缺乏特异性，多数患者仅仅以咳嗽或喘憋、呼吸困难等胸腔积液的临床表现为主诉入院，或在体检时发现肺部结节性病变，影像学表现缺乏特异性^[5]^。X线和CT平扫主要表现为肺外周的孤立性结节或分叶状肿块，肺部增强CT则多为轻到中度强化，与大部分支气管肺癌明显强化存在差异。同时黑色素瘤在磁共振成像(magnetic resonance imaging, MRI)上具有特征性T1WI高信号、T2WI低信号，具有诊断价值，但无法鉴别肺的原发或转移。与此同时，在血液生化检查方面，该病亦缺乏特异性，目前恶性黑色素瘤尚无特异的血清肿瘤标志物，即血清肿瘤标志物检查对诊断意义有限，在本例报道中也仅出现CA125升高。目前有文献指出，血清LDH升高与患者的预后相关，王亚丽等研究指出，血清LDH是影响恶性黑色素瘤患者总生存时间的独立预后因素，LDH正常者预后较好。最后对于此种疾病，病理诊断是金标准，文献^[5]^提示S100、HMB45和波形蛋白(Vimentin)是诊断黑色素瘤的特异性指标。本例患者胸部及腹部CT均有阳性结果提示，胸水涂片未见肿瘤细胞，但肺部结节术后病理明确诊断为黑色素瘤，免疫组化亦提示S100、HMB45和波形蛋白(Vimentin)均为阳性，支持恶性黑色素瘤诊断。患者全身皮肤检查颜色正常，未见皮疹、色素痣等，考虑本例黑色素瘤为胸膜原发。

### 2.3 治疗

目前为止，原发性胸膜恶性黑色素瘤的生物学行为不可预测，大多数患者从出现症状到病情进展的过程十分迅速，患者生存期短，预后极差。针对该病的治疗，目前美国国立综合癌症网络(National Comprehensive Cancer Network, NCCN)指南未给出明确的意见，主要是手术治疗辅以放疗、化疗，并结合相应的免疫治疗，病情晚期一般以个体化的综合治疗为原则。目前已知黑色素瘤对放疗、化疗均不敏感，但在某些特殊情况下，如骨转移、脑转移时，放、化疗仍是一种十分重要的治疗手段。在死亡原因方面，远处转移导致的多脏器功能衰竭是导致患者死亡的主要原因^[6]^。
